# Concept analysis of youth: understanding the plurality of subjects

**DOI:** 10.1590/0034-7167-2024-0002

**Published:** 2024-11-22

**Authors:** André Ribeiro de Castro, Moziane Mendonça de Araújo, Fabiane do Amaral Gubert, Marcos Venícios de Oliveira Lopes, Viviane Martins da Silva, Cristiana Brasil de Almeida Rebouças

**Affiliations:** I Universidade Federal do Ceará. Fortaleza, Ceará, Brazil

**Keywords:** Adolescent Health, Adolescent, Young Adult, Nursing, Concept Formation, Salud del Adolescente, Adolescente, Adulto Joven, Enfermería, Formación de conceptos

## Abstract

**Objective:**

To analyze the concept of “youth.”

**Methods:**

This is a concept analysis guided by the method proposed by Walker and Avant, operationalized through an integrative literature review. The search in scientific databases was carried out using the descriptors: youth; young; adolescence. To compose the literary corpus, 22 studies were selected.

**Results:**

Various factors were found in the antecedents that influence the separation of youth groups, contributing to the heterogenization of this population. The attributes include characteristics that comprise the formation of youth groups, especially the idea of shared experiences and social construction, as well as the consequences of the mentioned concept. After systematizing the variables, the analysis was conducted, highlighting the conceptions that influence youth.

**Conclusion:**

The study demonstrated the multifactorial complexity of the formulation of the concept of youth, highlighting various factors that contribute to this construction. For nursing, this conceptual field allows for an understanding of the population and effective engagement with this group.

## RESUMEN

**Objetivo:**

analizar el concepto de “juventudes”.

**Métodos:**

Se trata de un análisis de concepto, orientado por el método propuesto por Walker y Avant, operacionalizado mediante una revisión integrativa de la literatura. La búsqueda en bases de datos científicas se realizó utilizando los descriptores: juventud; joven; adolescencia. Para componer el corpus literario, se seleccionaron 22 estudios.

**Resultados:**

Se constataron, en los antecedentes, diversos factores que influyen en la separación de los grupos juveniles, influyendo en la heterogeneización de esta población. En los atributos, se integran características que comprenden la formulación de los grupos juveniles, sobre todo la idea de compartir vivencias y la construcción social, así como los consecuentes del referido concepto. Tras la sistematización de las variables, se procedió al análisis, destacando las concepciones que influyen en las juventudes.

**Conclusión:**

El estudio demostró la complejidad multifactorial de la formulación del concepto de juventudes, evidenciando diversos factores que componen esta construcción. Para la enfermería, este campo conceptual permite comprender a la población y actuar de forma efectiva junto a este grupo.

## INTRODUCTION

The conception of the life stage involving adolescents and young people has become naturalized as a phase encompassing the age-related transition common to individuals, often associated with the experience of conflicts and the transgression of social norms. This idea has been disseminated across various fields of knowledge, reducing individuals to a perspective constrained by age brackets that do not consider their relationships with the world^(1)^.

These ingrained conceptions are endorsed by the biomedical paradigm, which is anchored in a limited understanding of the individual based on the health/disease dichotomy. The demarcation legitimized by medical authority crystallizes the characteristics that link adolescents and young people to behaviors deemed appropriate and/or inappropriate by society as a whole^(2)^.

In addition to chronological division, historical context and experiences within the individual’s territory, as well as a range of specific experiences, influence characteristics and ways of acting, feeling, and thinking. In other words, an individual’s experiences make them different from their age group, considering subjectivity and the way they process events. Thus, age alone is not sufficient to define the multiplicity of attitudes present in a generation, nor to explain the differences among these individuals^(3)^.

An important reference in the field of youth studies is the understanding of the concept of “youths” in the plural, related to the recognition that individuals are influenced by the financial, economic, and cultural conditions in which they live^(4)^. Consequently, there is no single definition that can encompass the semantic field that youth can acquire; rather, there is a broad spectrum of perceptions about youth cultures related to different theories^(5)^.

In light of this, it is considered that there are multiple youths, understanding this category as temporal, dynamic, plural, and diverse, not defined solely by age, but by social, cultural, and economic context.

The conception of the concept of youths poses a challenge for researchers and institutions that deal directly with young people. In academia, for example, few studies provide this definition for an approach to young people and their respective perceptions, often failing to understand their experiences and expressions. Most studies discuss youths from the perspective of adults and institutions related to the lives of young people, without clearly defining the concept^(5)^.

The conceptual conflicts observed refer to the fact that many authors seek superficial criteria, such as age range, to define youth, failing to understand the plurality of youths as a more complex set of factors that permeate these individuals. This makes clarifying this concept an important research focus. This lack of understanding about the term motivates research to establish a conceptual synthesis for understanding the breadth of youth groups, enabling nursing to develop strategies that recognize the limitations and potential behind actions directed at these groups.

Clearly understanding the meaning of the concept of youths can lead to the recognition of these youth groups in different settings, enabling nursing to develop strategies that identify the limitations and potential behind actions. The nurse’s role in clinical practice should encompass both the individual and the collective being served. In this sense, it is necessary to study this concept, as it is still underutilized in nursing^(6)^.

Given the importance of understanding a concept, it is understood that defining, clarifying, evaluating, operationalizing, and subjecting concepts to theoretical and empirical evaluations are essential processes in advancing the knowledge of a field. The clearer the concept, the better the understanding and contribution it provides^(7)^.

## OBJECTIVE

To analyze the concept of “youths”.

## METHODS

### Ethical Aspects

As this study used public domain data and did not involve human subjects, there was no need for review by an Ethics Committee. However, the studies selected for the final sample were properly referenced.

### Study Design

This is a descriptive study that utilized the Walker and Avant Conceptual Analysis Model as a reference framework. Concept analysis involves describing a word or term, clarifying its use in the language, and elucidating how it is associated with or distinct from other related words or terms. The interest of this model lies in the actual and potential uses of words that convey conceptual meanings. The model consists of eight steps: selection of the concept; determination of the aims and purposes of the analysis; identification of all possible uses of the concept; determination of defining attributes; identification of a model case; identification of related, contrary, borderline, invented, and illegitimate cases; identification of antecedents and consequences; and definition of empirical referents^(8)^.

In this study, the following steps were followed: selection of the concept; determination of the aims of the conceptual analysis; identification of possible uses of the concept; identification of attributes, antecedents, and consequences. The selection of the concept should reflect the topic or area of greatest interest. Determination of the aims of the conceptual analysis refers to the purpose of the conceptual analysis intended. Identification of the possible uses of the concept involves a literature search to understand how the concept in question is being focused on or applied. Determination of the critical attributes involves presenting the attributes or groups of attributes frequently associated with the concept, allowing for a better understanding of these, distinguishing it from similar or related concepts. Antecedents are events that occur before the identification of the event itself, contributing to its occurrence. Consequences refer to a set of factors triggered once the phenomenon in question is confirmed^(8)^.

It is emphasized that some steps of the Concept Analysis Model are equivalent and occur simultaneously with the steps of the integrative literature review, such as the selection of the concept; determination of the aims of the conceptual analysis; and identification of the possible uses of the concept^(8)^. Initially, for the concept analysis, the concept of youths was selected. In this stage, the aims were to conduct a concept analysis of the term youths to identify its uses (definitions), critical attributes, possible antecedents, and consequences. The main objective was to find an initial structure for the concept that would allow for a clear definition and establish causal elements (antecedents) and possible expressions and outcomes associated with the occurrence of that concept. Furthermore, the work aims to present a more specific discussion of the elements found to minimize the need for other steps.

To carry out the proposed steps, an integrative literature review was conducted following the five suggested steps: identification of the theme or question of the integrative review, sampling or literature search, evaluation of the included studies, interpretation of the results, and synthesis of the knowledge evidenced in the articles analyzed in the review^(9)^.

To formulate the research question, the PICO strategy (an acronym for Patient, Intervention, Comparison, Outcomes) was used^(10)^. The first element of the strategy (P) refers to adolescents, young people, and youths; the second element (I) refers to the life stage experienced; and the fourth element (O) concerns social groups. In this study, the comparison element (C) was not used because this research does not aim to compare interventions. The use of the PICO strategy proves efficient in effectively retrieving evidence, and some databases have an interface for the direct insertion of the four PICO components, such as MEDLINE/PubMed. What are the attributes, antecedents, and consequences related to the concept of youths identified in the literature?

After identifying the main concepts in the literature, the next step is to determine the defining attributes, which involves gathering the attributes most frequently associated with the concept. The idea of an attribute encompasses that by which a substance is known or, from the theory of attributes, that which expresses the essence of the substance or concept. In the stage of identifying the antecedents and consequences of the concept, the verification of events that occur before the phenomenon of interest (antecedents), which are necessary for its occurrence, takes place, as well as those that happen after the concept and result from the presence of the phenomenon (consequences)^(8)^.

### Study Period and Location

The search was conducted via the Virtual Health Library (VHL), which included the Latin American and Caribbean Health Sciences Literature (LILACS), the International Literature in Health Sciences (MEDLINE), the Nursing Database (BDENF), and the Spanish Bibliographic Index in Health Sciences (IBECS).

The combination of Health Sciences Descriptors (DeCS) used included: youth; young; adolescence. The descriptors were combined using the boolean operator AND, with the key combination being: “youth” AND “youths” AND “young” AND “adolescence.” The search was performed in pairs by the researchers and was conducted in May and June 2023. Data were collected using a structured instrument, which included the characterization of the studies, attributes, antecedents, and consequences identified in the literature regarding the concept of youths.

### Inclusion and Exclusion Criteria

Studies included were those in Portuguese, Spanish, or English, selecting original articles, theoretical papers, reflective articles, and review articles available in full. Exclusion criteria included opinion articles, letters to the editor, dissertations, and theses, as gray literature is not controlled by scientific or commercial publishers. Articles that did not contribute to a better understanding of the theme in question were also excluded. The temporal restriction was set from 1990, as this is when the term was incorporated.

Initially, 106 articles were found. Among the articles investigated, 24 were excluded because they were unavailable texts or theses and dissertations. Thirteen were excluded as duplicates, leaving 69 for title and abstract screening. After this stage, four were eliminated because they did not relate to youth/youths in the titles or abstracts. Thus, 65 articles were read in full, with 43 excluded for not having a direct relation to the conceptualization of youths. Finally, 22 articles were selected. Of these, 19 were indexed in the LILACS database, two in the BDENF database, and one in the IBECS database.

### Analysis of Results

Data collection was guided by a form developed by researchers with expertise in working with adolescents and youths, as well as literature reviews to extract the following variables: database; language; year of publication; country where the study was conducted; study objective; methodological design; and central concepts on the topic. The data were organized and digitized in spreadsheets using Microsoft Excel® 2017. A descriptive synthesis of the findings was conducted to clarify the concepts related to the data found.

## RESULTS

**Figure 1:**
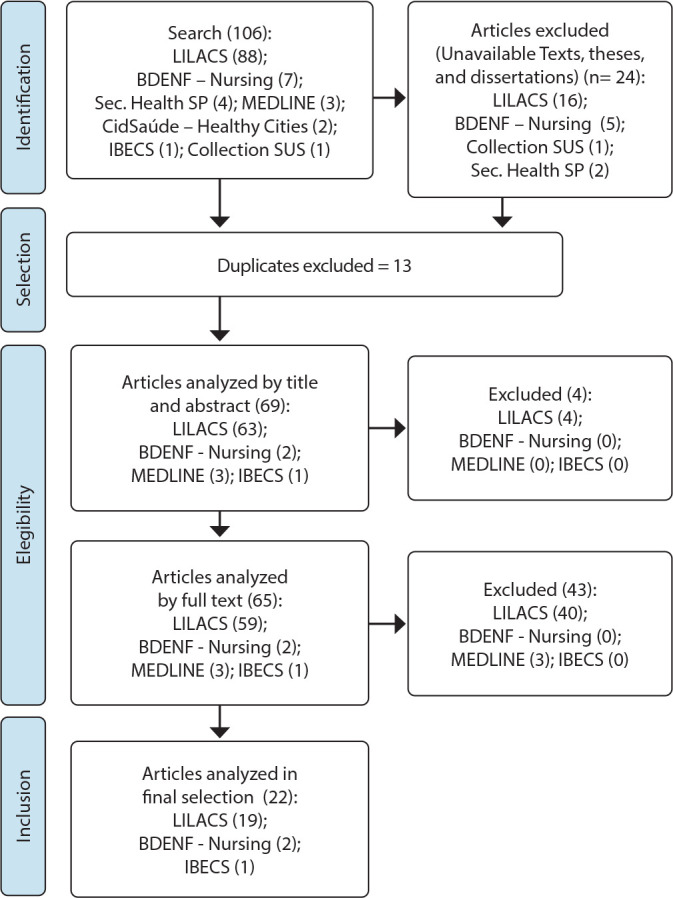
Flowchart of the Study Selection Process for Integrative Review, Adapted from PRISMA-ScR, 2024

The selection of articles was conducted through searches in the databases available in the Virtual Health Library (VHL), resulting in a total of 22 articles. The process followed during this search is summarized in Figure 1.

Among the sample of studies, presented in the Chart 1, it was found that the majority were developed in the field of Psychology (n=9), followed by Sociology (n=4), Nursing (n=4), and Physical Education (n=2). Other areas with fewer studies included Philosophy (n=1), Occupational Therapy (n=1), and Social Work (n=1). There was no significant exploration of the concept of youths in the field of Nursing, reaffirming the need to define it.

**Chart 1:** Description of Studies Included in the Integrative Review by Title, Authors/Year of Publication, Level of Evidence, Design, Number of Participants and Setting, Palliative Care Interventions, and Outcomes, 2024

ID	Titles	Authors/Year of Publication	Journal/Database	Objectives	Synthesis of the Concept of Youth/Youths
A1	Reflexões acerca da prática clínica do enfermeiro junto às juventud (6)^(6)^	Castro Júnior AR, et al., 2020	Cultura de Los Cuidados/IBECS	To reflect on the clinical practice of nurses working with youths.	Understanding the various factors that involve the life of a young person necessitates the pluralization of the term youth, thus using “youths” to encompass the diverse realities in which these youth groups find themselves.
A2	Percursos metodológicos para a apreensão de universos de adolescentes e jovens: um enfoque sobre a questão das drogas(11)^(11)^	Pereira PE, Malfitano APS, 2012	Journal of Human Growth and Development/LI LACS	To investigate the perspective of pedagogical agents on the leisure practices of adolescents serving socio-educational measures in the city of Campinas-SP, Brazil.	There are different ways of experiencing youth, depending on the social, historical, economic, and political context; gender, social class, ethnicity, and place of residence (rural or urban, central or peripheral). Thus, there are multiple youths.
A3	Mapa das juventudes de Santo André, SP: instrumento de leitura das desigualdades sociais^(12)^	Yonekura T, et al., 2010	Revista Saúde Pública/LILACS	To investigate the perspective of pedagogical agents on the leisure practices of adolescents serving socio-educational measures in the city of Campinas-SP, Brazil.	The perspective of public health understands youth through the categories of social class and generation, interpreting the contemporary reality of young people through the lens of class condition. There are, therefore, various youths, and although they share the generational condition, they experience it differently depending on their family’s and society’s context.
A4	Consulta de enfermagem no cuidado ambulatorial às juventudes^(13)^	Castro Junior AR, et al., 2019	Revista de Enfermagem da Universidade Federal de Pernambuco/BD ENF	To identify the scientific evidence regarding nursing consultations in outpatient care for youths.	In the health field, there is a conceptual imprecision regarding adolescence, young people, and youth, starting primarily from the arbitrariness in defining age limits. When considering legal documents about adolescence and youth, it is evident that even these do not present unified definitions of the life stage defined by these terms.
A5	*Género y juventudes Argentinas: ¿de qué estamos hablando cuando hablamos de “el problema del embarazo adolescente”?* ^(15)^	Duran, ABD, Muro MM, 2020	Sexualidad, Salud y Sociedad Revista Latinoamericana/ LILACS	To examine the issue of teenage pregnancy as a problem through a historical journey, utilizing journalistic sources and a legal corpus.	The concept of “youth” in the plural emphasizes that there are various ways of being young, acknowledging that it is far from a homogeneous phenomenon. This perspective considers different adolescents influenced by various social processes and diverse social markers of difference, such as social class, gender, and race.
A6	Juventudes e participação: compreensão de política, valores e práticas sociais^(16)^	Mesquita MR, et al., 2016	Psicologia e Sociedade/LILA CS	To investigate the perspective of pedagogical agents on the leisure practices of adolescents serving socio-educational measures in the city of Campinas-SP, Brazil.	Youths are both a social condition and a type of representation that no longer pertains only to a particular life stage associated with a specific age group. It is emphasized that the group cannot be characterized as homogeneous merely because its members share the same age.
A7	*Contextualización teórica al tema de las juventudes: una mirada desde las ciências sociales a la juventud^(17)^*	Alvarado SV, Posada JEM, Gaviria DAM, 2009	Sexualidad, Salud y Sociedad Revista Latinoamericana/ LILACS	To present an approach to contemporary sociological theories based on the theme of youth, utilizing concepts through which the social sciences, particularly sociology, have understood youth dynamics.	The understanding of youths involves a dialogue of discourses that adhere to specific space/time conditions. It is pertinent to propose a possible thematic path that reflects on the symbolic imaginaries that produce and are produced by perceptions and self-perceptions of youths, thus transcending mere psychoevolutionary localization within a certain age group.
A8	A percepção das juventudes a respeito dos seus processos de identidades e suas apropriações para além do jogador Neymar^(18)^	Feltes AF, Sanfelice GR.	Movimento/LIL ACS	To analyze and interpret the relationship between identity construction and youths’ perceptions regarding the appropriation of the figure of the soccer player Neymar.	Different social groups use representation to construct their own identities and the identities of other groups, even if this identification is temporary. Youths build their identity traits through references in communities and do not want to be confined to stereotypes.
A9	Juventudes e medida socioeducativa: o olhar dos agentes pedagógicos sobre as práticas de lazer^(20)^	Tretin DFL, et al., 2018	Pensar a Prática/LILACS	To investigate the perspective of pedagogical agents on the leisure practices of adolescents serving socio-educational measures in the city of Campinas-SP, Brazil.	It is understood that youth is the stage of life where everyday experiences and situations have a significant influence on the individual’s overall development. Moreover, the term “youths” characterizes a social stratum with certain features: “a tangible reality that includes sex, age, race, and phases, shaped by their material and social conditions.”
A10	Juventude em Foco: Representações Sociais da Juventude na Folha de S.Paulo^(21)^	Rezende JWR, Espindula DHP, 2020	Psicologia: Ciência e Profissão/LILAC S	To investigate the social representations of youth in articles from the newspaper Folha de S. Paulo.	The conception of youths presents the idea of the existence of a plurality of youths, noting that the media refers to this period through a set of aspects that are not only biological but also cultural and social, including life experiences and shared moments.
A11	Jovens, imagens de si e a cidade: discursos em movimento^(22)^	Levitan D, et al., 2009	Journal of Human Growth and Development/LI LACS	To understand the relationships between youth and the city, as well as the meanings that characterize this stage of life for some young people.	The use of “youths” in the plural, rather than “youth” in the singular, highlights the differences and inequalities that cross this condition. The plural form indicates the recognition that youth is a condition present in various social groups, but it can be interpreted differently by each, emphasizing the diverse ways of experiencing this stage of life.
A12	*Hacia un entendimiento del aprendizaje en entornos digitales-Implicaciones para la educación^(23)^*	Morales-Barreira MCM, 2018	Sexualidad, Salud y Sociedad Revista Latinoamericana/ LILACS	To analyze the characteristics of learning based on the digital competencies that higher education students develop in non-school sociocultural digital environments.	The pluralization of the term implies an epistemological perspective of youth, based on recognizing heterogeneity as the axis of multiple subjective constructions that cannot be subsumed into a single, closed identity. From this viewpoint, the category of youth has two important implications: the erosion of the supposed identity of youth and the necessity to historicize the production of youthful subjectivities.
A13	Encontros com a diferença na formação de profissionais de saúde: juventudes, sexualidades e gêneros na escola^(24)^	Silva CG, Borba PLO, 2018	Saúde e Sociedade/LILA CS	To discuss how gender and sexuality are interpreted by high school students, drawing on reflections from the university extension project “Youth and Funk in the Baixada Santista.”*”*.	In the discussions held, the focus is on identifying and understanding the intersections among the social markers of difference that constitute young people. As a methodological and analytical tool, intersectionality allows for the decoding of irregularities and regularities inscribed in the subjects of youths within the territories they inhabit.
A14	Os projetos de vida dos jovens da maior favela carioca, a Maré^(26)^	Santos SRR, 2018	Revista de Psicologia/LILA CS	To design a diagram of the knowledge-power dynamics surrounding youth in the field of health.	Although “young person” and “youth” are often treated as synonyms, “young person” always refers to the individual, while “youth” refers to the time of the individual. Youth is not only a period of changes; it is also a phase of planning and future construction.
A15	O lugar das juventudes nas produções científicas da Estratégia Saúde da Família: uma revisão sistemática de literatura^(28)^	Benício LFS, et al., 2019	Textos e Contextos (Porto Alegre)/LILACS	To design a diagram of the knowledge-power dynamics surrounding youth in the field of health.	In capitalist society, youth is constructed from a developmental perspective, positioning them as beings in progress, whose behaviors result from physical and psychological changes. Within the various youths, issues of violence and criminality are added, producing the notion of the “dangerous youth.”
A16	*Miradas docentes sobre los jóvenes y las jóvenes como «otros indeseables» en secundarias universitarias en La Plata* ^(30)^	Piero ED, 2019	Revista Latinoamericana de Ciencias Sociales, Niñez y Juventud/LILAC S	To present teachers’ views on young people as “undesirable others” in university-affiliated high schools in La Plata.	In more biological or demographic propositions, young men and women are defined based on biological age. In contrast, recent research has highlighted the emergence of a new “youth condition” within a context of profound social, economic, and cultural changes.
A17	Primaveras e jovens tardes: a memória saudosa da mocidade/juventude na música popular brasileira^(31)^	Nascimento ARA, et al., 2010	Memorandum/LI LACS	To identify, in correlation, the elements nostalgically remembered from the youth period present in the lyrics of Brazilian songs composed and/or recorded since 1927.	The existence of diverse representations of youth coexisting in the same society is acknowledged. It must be considered that the sharing of a particular meaning does not necessarily imply the perception of “youth” as a single, uniform category.
A18	Produção social, histórica e cultural do conceito de juventudes heterogêneas potencializa ações políticas^(32)^	Trancoso AER, Oliveira AAS, 2014	Psicologia e Sociedade/LILA CS	The social, historical, and cultural production of the concept of heterogeneous youths enhances political actions.	Youths are “social constructions, ‘age classes’” that, “despite having a biological material basis, have various historical representations.” The term youths does not only encompass the bodily changes and hormones associated with age but also marks the epistemological roots of culture.
A19	Programa Nacional de Inclusão de Jovens: possibilidades e Contribuições na Perspectiva dos adolescentes participantes^(33)^	Campos CCA, Paiva IL, 2018	Fracal/Revista de Psicologia/LILA CS	To discuss the socioeducational process of the National Youth Inclusion Program, in the Projovem Adolescente (PJA) modality, using the perspective of adolescent participants from the city of Natal/RN.	There is a dichotomy regarding the conception of youth. Given the diversity of existential situations that affect individuals at this stage of the life cycle, it has become increasingly important to consider the idea of youth and adolescence in a plural sense - youths and adolescences.
A20	Uma caderneta que produz ‘saúde´ e ‘adolescentes´^(34)^	Hüning SM, Luz Valeska CM, 2011	Arquivos Brasileiros de Psicologia/LILA CS	To critically examine the Adolescent Health Booklet produced by the Ministry of Health.	When studying the life of adolescents, they can be perceived and studied as a diverse social group, identified by different youth cultures. The term youths, in the plural, seeks to understand complexity and heterogeneity.
A21	Uma Promessa Civilizatória Perversa: as políticas públicas e juventudes na era neodesenvolvimentista^(35)^	Scherer GA, Gershenson B, 2016	Textos e Contextos (Porto Alegre) LILACS	To present the context of social (in)protection experienced by Brazilian youths, revealing the main expressions of social issues that impact this social segment.	The term “youths” in the plural is considered a way to indicate its multiplicity, based on the premise that it is necessary to take into account a series of particularities that compose this segment, such as race, social class, territory, and other elements.
A22	Jovens em webrádio: representações sociais sobre papilomavírus humano^(36)^	Araújo AF, et al., 2019	Revista de Enfermagem da Universidade Federal de Pernambuco/BD ENF	To learn about the social representations of school-aged youth regarding the human papillomavirus through a webradio.	Youths develop in both personal and social perspectives, as subjects of rights and as actors with a special role in transforming reality. They are centrally marked by processes of development, social integration, and the definition of diverse identities.

Regarding language, there was a predominance of research published in Portuguese (n=18), as most of the sample was produced in Brazil (n=18). There were also articles in Spanish (n=4), distributed among the countries: Colombia (n=2) and Argentina (n=2), demonstrating the international use of the term.

**Figure 2:**
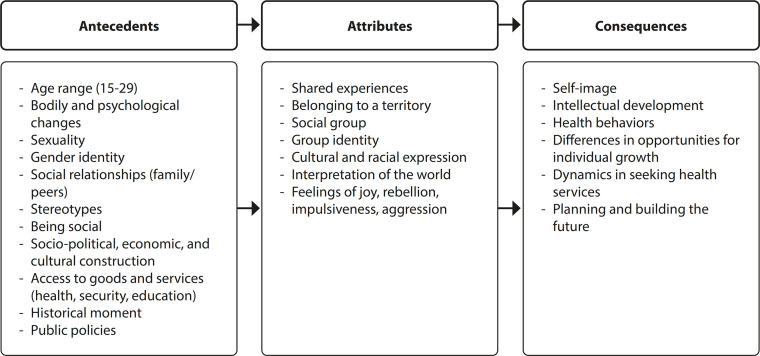
Antecedents, Attributes, and Consequences of the Concept of Youths, 2024

Considering methodological aspects, the qualitative approach prevailed, especially exploratory studies (n=10), followed by reflective studies (n=6), bibliographic reviews (n=3), and a documentary study (n=1). There was a small representation of quantitative articles (n=1), corresponding to an ecological study (n=1). The elements addressed according to phenomenon, antecedents, attributes, and consequences are presented in categories and subcategories of analysis, as shown in Figure 2.

## DISCUSSION

This analysis identified various components that define youths, highlighting elements that compose antecedents, attributes, and consequences. It is believed that this is linked to the broad understanding of what youths are, which has been evolving over time, especially within each area of knowledge. As outlined in the synthesis, Nursing still discusses this topic infrequently, often citing sources from the social sciences. Among the studies analyzed in this research, most associated youth with age-related conceptions and other more physical elements, while youths encompass the relationships these young individuals have with various life components. Therefore, there is a need to implement more elaborate concepts about youths, supporting manuscripts that discuss nurses’ actions in clinical practice.

The studies highlighted indicate that the phenomenon of youths cannot be predominantly related to the age-based division model. In this sense, the discussion of the findings succinctly addresses the elements that make up the antecedents, attributes, and consequences of the term youths, thus relating these components to the authors’ understanding of definitions and approaches in the lives of youths.

### Antecedents of the Concept “Youths”

The antecedents encompass the determinants of the concept, what precedes the event and the emergence of the outcome. The results aim to show that the concept of youths is the result of situations related to the experiences of youth groups, leading to a plural understanding of youth.

The construction of the concept of youths involves a social understanding that includes various factors. It defines a young person as an individual aged between 15 and 29 years, understanding that this conceptualization encompasses changes in the biological field with the maturation of physical characteristics, the expression of sexuality, and sexual experiences regarding sexual orientation and gender identity. These expressions of sexuality are not limited to the sexual act but also include the ways youths relate to each other. This period also involves intense psychological changes, the understanding of oneself as a subject, and personal conflicts caused by these changes and maturation^(11)^.

This period of change in young people leads to questions about body transition and relationships. Alongside these changes, there is a search for identity, based on family experiences as well as relationships with friends (peers). These relationships influence the formation of bonds and the search for acceptance in social groups where they feel they belong^(12-13)^.

Understanding the concept of youths includes the multiple references that affect the experiences of young people. Issues of gender, social class, and ethnicity are frequently discussed in relation to this definition. The terms young person, youth, and their plural forms can also be rooted in various stereotypes that tend to incorrectly define this group, often labeling them as a “problematic figure”^(14)^.

This multiplicity of factors and experiences within the same population segment reinforces the dynamic and ever-evolving concept of youth, giving rise to the pluralization of the term as “youths.” It is impossible to define this word without considering the entire social, political, and economic construction that impacts these individuals^(11)^.

In the definition of youths, it is essential to understand social inequalities and how this factor impacts the population, particularly regarding access to goods and services such as health, education, and security. This dynamic facilitates the recognition of heterogeneity within the group, as social dynamics determine where and how people live and influence the relationships and opportunities they access^(15)^.

Understanding social dynamics is crucial for defining the concept of youths, as is understanding the historical context in which this definition is considered. The concept of youth has been widely discussed throughout history, always distancing the concept from any homogenizing pretensions about the population^(12,15)^.

The different forms of contact and political participation can determine how this population is viewed within a territory (country, state) and influence the actions directed toward these young people concerning health, security, sports, work, and other aspects that affect their growth opportunities. It is important to recognize the existence of multiple forms of youth organization and their social integration into groups undergoing constant transformations to define what youths are and the scope of this concept^(16)^.

### Attributes of the Concept “Youths”

The attributes encompass the properties of the concept, being elements that express the main characteristics of the construct. These attributes can reveal themselves as social relationships, environmental influences, among other characteristics, depending on the concept and the central object. Here, the meanings of the pluralization of the term and its collective repercussion are presented. The identity process is identified as the expression of what unites individuals according to individual and collective choices, giving meaning to that collective. Thus, the understanding of the concept of youths is increasingly tied to the sharing of experiences that place the individual within a territory of experimentation, giving them the notion of belonging to that place and group^(11)^.

From the findings, it is understood that youths are forged from social and generational contexts (the historical time in which they are inserted); however, discussions strongly emphasize the sharing of experiences. The various youths, even within the same historical period, have their own experiences and organize themselves into different youth cultures, identifying with different groups^(12,17)^.

The identities of youths are formulated through experiences as well as collective practices of shared interests, culminating in the idea of “being together” and “being part of”^(18)^. With the collective formulation of the meanings that unite social groups, which includes the idea of group identity, it is common to find affinity through the union of common cultural and racial expressions. Being close to someone who identifies with them provides a sense of belonging to the individual. Although there are delimitations for the condition of youth, the experience of being young requires pluralization^(19-20)^.

According to the idea of collective construction and all the cultural expressions that involve young people, as highlighted in the texts, the concept of youths goes beyond being limited to a phase of human development and encompasses a way of life, an identification within a group that connects through a similar interpretation of the world^(21)^.

From the understanding of different ways of relating throughout life, according to particular socio-historical contexts, the plural conception of youth breaks the universal and rigid logic that does not encompass all the experiences of this group. The plural serves to remind us of the differences and inequalities that cross this condition. The way of understanding the world within different social groups contains unique meanings, emphasizing the different modes of living or how these experiences impact the feelings shared by the group^(22)^.

It is possible to identify, through the literature findings, the pluralization of youths from an epistemological perspective, centered on subjective constructions that cannot be reduced to age determination. Ignoring the different conditions and ways of being young entails excluding everything that does not fit into the stereotypical identity of youth, thereby disregarding meanings and feelings^(23)^.

This group context involves the construction of a social network that produces subjectivities, connecting individuals and allowing for the identification of feelings such as the joy of being young, the rebellion associated with transformations, and the impulsiveness of age. Thus, it encompasses a relational dynamic of individuals who create connections and support within the context of their experiences^(24)^.

### Consequences of the Concept “Youths”

The consequences highlight the outcomes of the analyzed concept, revealing how the concept is expressed and what this phenomenon entails for the population, in this case, the youths. The main consequences identified include self-image, intellectual development, health behaviors, differences in opportunities for individual growth, dynamics in seeking health services, and planning and building the future.

The self-image discussed in the findings reveals the way of being and living, prevailing values, and the relationships established in society. It is formed through the daily experiences of young people, a condition that stems from individual arrangements and constitutes an understanding of oneself based on the fusion of social standards and constructed relationships. The crucial importance of self-image in identity construction is emphasized^(25)^.

The construction of youth identity is highlighted in the literature as intrinsic to intellectual development. This development occurs through personal and social experiences, determined by skills developed from childhood to adulthood. In this sense, the family, school, and social context in which young people are embedded play fundamental roles^(26)^.

According to the social context of youth, as evidenced in the realities presented in the articles, different opportunities can be offered within socio-economic contexts. The interaction with the environment and its influences can guide practices and choices that interlink life development and opportunities for these young people regarding work, education, and social conditions. Not everything is determined by social interaction, but it connects the way experiences are produced and how opportunities and adversities are faced. There are stigmas associated with young people from lower socio-economic backgrounds, mostly Black and poor, living in urban peripheries, labeled as violent and delinquent, forging inequities that place this youth group in different conditions with fewer opportunities for life improvement^(27)^.

The articles identify that ways of living, the idea of the “invulnerable” youth, and the formation of influences are important factors to consider when observing the search for health services. The formation of youth influences the low utilization of services, and when sought, they encounter resistance and difficulty in addressing youth demands, considering the health behaviors of this group, which can be safe or risky. Issues such as bodily changes, intellectual development, personal identity formation, and peer interactions influence the first sexual experience, and alcohol and drug use, with alcohol considered a risk factor for unsafe sexual practices and the acquisition of sexually transmitted infections^(28)^.

In the various social contexts in which young people are involved, youth is highlighted in the literature as a phase of planning and world-building, considered a transitional stage to adulthood. In the Medieval Ages, this phase was marked by rituals. Today, this process occurs through the social groups to which young people belong, such as family, school, church, and circles of friends^(24,28-29)^.

The literature also highlights that friendship, the possibility of meeting, and sharing affections produce and enhance the sense of belonging to a group, reinforcing communal, cultural, and political ideals, and enabling the construction of common projects in the face of numerous challenges. These values drive practices and help them resist difficulties with a sense of overcoming, renewal, and learning^(16,30-32)^.

It is important to note that the values shared by young people are diverse. However, with the desire for transformation as a major value, they build notions of collectivity and community that help them recognize themselves as important agents of change, necessary to fulfill the aspirations of a better society. Additionally, they share more subjective values, such as friendship and pleasure, which can become crucial for group organization and the process of political subjectivation^(16,33-35)^.

As consumers of goods and services, particularly in health, they face access difficulties, with their needs often being reduced or overlooked. In the health field, there are still gaps in strategies that encompass care that understands the representations of youths and their particularities, posing challenges to services and professionals^(6,29)^. Filling these gaps can enhance the understanding of clinical care practices for this population, addressing their needs and considering the limitations and possibilities for this group^(20,29)^.

Given the above, the concept of youths includes expanding the concept of young people, considering the bodily and psychological changes that occur between the ages of 15 and 29, but especially the phenomena responsible for personality development, such as sexuality, gender identity, and social relationships.

The construction of youths involves the nuances of social relationships, whether with family or peers, influencing how the individual constructs the idea of being social and their relationships. Association with these groups often brings stereotypes that solidify the image of young people. However, the understanding of the concept is still linked to the social, political, economic, and cultural construction in which the young person lives and which directly influences access to goods and services (health, security, education).

Youths organize themselves into groups with common characteristics, often united by shared experiences that are influenced by the historical moment and how it affects their lives. This includes the implementation of public policies and the understanding of what it means to be plural youths.

Finally, youths are conceptualized as a social group with their own unique identity, where social, cultural, and racial issues influence their interpretation of the world, the sharing of experiences, and their sense of belonging to a territory. This group is characterized by feelings of joy, rebellion, impulsiveness, and aggressiveness.

### Study Limitations

This research has limitations that must be considered. Initially, this conceptual analysis included only studies available electronically in certain databases, which may have resulted in the exclusion of research that addresses other perspectives on youths. Another limitation of the study is the exclusive use of a single portal for data retrieval, which may restrict the scope of the research and limit the variety of available information.

### Contributions to the Field

The findings promote an understanding of the broad comprehension of youth groups and the potential strategies that nurses can adopt. Concept analysis allows for the inclusion of the phenomenon in nursing team training programs. Expanding the understanding of this population group helps nurses comprehend different realities, enabling the multidisciplinary team to work from health promotion, harm reduction, and mitigation of adverse outcomes, recognizing that various strategies are necessary to reach this audience. The effort to analyze the concept is the first step in understanding the individuals and social phenomena involved in their experiences, thereby broadening the scope of nursing practice.

## FINAL CONSIDERATIONS

Understanding the antecedents, attributes, and consequences helps in comprehending the concept of youths in the health field, especially for nursing. This understanding delineates important information about the formation of these youths and youth groups, expanding comprehension beyond the age range and considering the processes that influence the formation of these youth cultures. Given the considerations presented, the analyzed articles reveal a panorama of the concept of “youths,” confirming the complexity arising from the multifactorial origin of the phenomenon under study. It is also noted that despite the widespread use of the term youths, there is no consensus in the literature, highlighting divergent definitions that do not adequately support nursing care for youths.

The concept analysis evidenced by this study can support nursing care for youths, considering the plurality and heterogeneity of this group, aiming at actions targeted to the real needs of young people, making the care qualified and effective. Understanding the concept provides an opportunity to act with this young population in different contexts and realities, offering theoretical support to nurses in this role.
