# Supplementary material for: Concept analysis of youth: understanding the plurality of subjects
Source: Rev Bras Enferm. 2024 Nov 22;77(4):e20240002. doi: 10.1590/0034-7167-2024-0002 (PMC11654225; doi:10.1590/0034-7167-2024-0002)
Supplement: Translation

# Translation: Análise de conceito sobre juventudes: compreensão da pluralidade dos sujeitos

**DOI:** 10.1590/0034-7167-2024-0002pt

**Authors:** André Ribeiro de Castro, Moziane Mendonça de Araújo, Fabiane do Amaral Gubert, Marcos Venícios de Oliveira Lopes, Viviane Martins da Silva, Cristiana Brasil de Almeida Rebouças

**Affiliations:** I Universidade Federal do Ceará. Fortaleza, Ceará, Brasil

**Keywords:** Saúde do Adolescente, Adolescente, Adulto Jovem, Enfermagem, Formação de Conceito

## Abstract

**Objetivo:**

analisar o conceito de “juventudes”.

**Métodos:**

Trata-se de análise de conceito, orientado pelo método proposto por Walker e Avant, operacionalizada mediante revisão integrativa da literatura. A busca em base de dados científicos foi efetivada, utilizando-se dos descritores: juventude; jovem; adolescência. Para compor o corpus literário, elencaram-se 22 estudos.

**Resultados:**

Constataram-se, nos antecedentes, diversos fatores que influenciam a separação dos grupos juvenis, influenciando a heterogeneização dessa população. Nos atributos, integram-se características que compreendem a formulação dos grupos juvenis, sobretudo a ideia de compartilhamento de vivências e construção social, bem como os consequentes do referido conceito. Após a sistematização das variáveis, procedeu-se à análise, destacando as concepções que influenciam as juventudes.

**Conclusão:**

O estudo demonstrou a complexidade multifatorial da formulação do conceito de juventudes, evidenciando diversos fatores que compõem essa construção. Para a enfermagem, esse campo conceitual permite compreender a população e atuar de forma efetiva junto a esse grupo.

## INTRODUÇÃO

A concepção da etapa de vida que envolve os adolescentes e os jovens torna-se naturalizada como etapa que abrange a transição etária relacionada ao ciclo da vida comum aos sujeitos, sendo, muitas vezes, relacionada à experimentação de conflitos e transgressão às normas sociais. Essa ideação vem sendo disseminada por vários campos do saber, reduzindo os sujeitos a uma visão delimitada por faixas temporais que não compreendem as relações desse sujeito com o mundo^(1)^.

Essas concepções enraizadas estão referendadas pelo paradigma biomédico, ancorado em uma compreensão reduzida do sujeito referente ao binômio saúde/doença. A demarcação legitimada pela autoridade médica cristaliza as características que ligam os adolescentes e os jovens aos comportamentos considerados adequados e/ou inadequados para o conjunto da sociedade^(2)^.

Além da divisão cronológica, a localização histórica e as vivências no território daquele sujeito ou do grupo, bem como uma gama de experiências específicas influenciam características e modo de agir, sentir e pensar. Ou seja, as vivências desse sujeito o tornam diferente do grupo etário a que pertence, considerando a subjetividade e a forma de processar os acontecimentos. Assim, a faixa etária sozinha passa a não ser suficiente para definir a multiplicidade de posturas que se apresentaram em uma geração, nem explicar as diferenças entre esses sujeitos^(3)^.

Importante referência no campo das juventudes é a compreensão do conceito “juventudes” no plural, relacionada ao entendimento de que o sujeito tem influência das condições financeiras, econômicas e culturais em que vive^(4)^. Deste modo, não há definição única que possa abranger o campo semântico que o jovem pode adquirir, o que há é um repleto campo de percepções sobre as culturas juvenis que são relacionadas às diferentes teorias^(5)^.

Diante disso, considera-se que há jovens e juventudes, compreendendo essa categoria como temporal, dinâmica, plural e diversa, que não se define apenas etariamente, mas considerando o contexto social, cultural e econômico.

A concepção do conceito juventudes confere desafio para pesquisadores e instituições que lidam diretamente com os jovens. No meio acadêmico, por exemplo, são identificadas poucas pesquisas que trazem essa definição para uma abordagem dos jovens e das respectivas percepções, muitas vezes, não compreendendo experiências e manifestações. Grande parte das produções discorre sobre as juventudes a partir da visão de adultos e instituições ligadas à vida dos jovens que não trazem clara a definição do conceito^(5)^.

Os conflitos conceituais observados se referem ao fato de muitos autores buscarem critérios superficiais, como a faixa etária, para definir jovem/juventude, não compreendendo a pluralidade das juventudes como conjunto mais complexo de fatores que permeiam esses sujeitos, tornando a clarificação deste conceito importante ponto de pesquisa. Essa falta de compreensão sobre o termo motiva a pesquisa a fim de estabelecer uma síntese conceitual para o entendimento da amplitude da compreensão sobre os grupos das juventudes, possibilitando à Enfermagem a elaboração de estratégias que permitam conhecer as limitações e as potencialidades por trás das ações voltadas a esses grupos.

Conhecer com clareza o significado do conceito juventudes pode representar o reconhecimento destes grupos juvenis em distintos cenários, possibilitando à Enfermagem a elaboração de estratégias que permitam conhecer as limitações e as potencialidades por trás das ações. A atuação do enfermeiro na prática clínica deve compreender o sujeito e o coletivo que se atende. Neste sentido, compreende-se a necessidade de se estudar esse conceito por entender que este ainda é pouco utilizado na Enfermagem^(6)^.

Diante da relevância de se compreender um conceito, entende-se que definir, esclarecer, avaliar, operacionalizar e submeter aos conceitos as avaliações teóricas e empíricas são processos essenciais no avanço do conhecimento de um campo do saber. Quanto mais claro o conceito, melhor o conhecimento e a contribuição do mesmo^(7)^.

## OBJETIVO

Analisar o conceito de “juventudes”.

## MÉTODOS

### Aspectos éticos

Por se tratar de estudo que utilizou dados de domínio público e não envolveu seres humanos, não houve necessidade de apreciação por Comitê de Ética em Pesquisa. Porém, os estudos selecionados para a amostra final foram devidamente referenciados.

### Desenho do estudo

Trata-se de estudo descritivo, no qual foi utilizado como referencial o Modelo de Análise Conceitual de Walker e Avant. A análise de conceito confere a descrição de uma palavra ou termo, cujo uso no idioma é explicitado juntamente com a elucidação de como se associam ou não a outras palavras ou termos relacionados. O interesse desse modelo está nos usos reais e possíveis de palavras que transmitem significados de conceito. O referido modelo consta de oito etapas: seleção do conceito; determinação dos objetivos e finalidades da análise; identificação de todos os usos possíveis do conceito; determinação dos atributos de definição; identificação de caso/modelo; identificação de casos/limite relacionados, contrários, inventados e inapropriados; identificação de antecedentes e consequentes; e definição de referenciais empíricos^(8)^.

Neste estudo, seguiram-se as etapas: seleção do conceito; determinação dos objetivos da análise conceitual; identificação dos possíveis usos do conceito; identificação dos atributos, antecedentes e consequentes. A seleção do conceito deve refletir o tópico ou a área de maior interesse. Determinação dos objetivos da análise conceitual se refere à finalidade da análise conceitual que se pretende realizar. A identificação dos possíveis usos do conceito consiste na busca na literatura para se ter uma ideia de como o conceito em questão está sendo enfocado ou aplicado. A determinação dos atributos críticos consiste em apresentar os atributos ou grupo deles que são frequentemente associados ao conceito, possibilitando maior compreensão destes, diferenciando-o de conceitos similares ou relacionados. Antecedentes são eventos ocorridos antes da identificação do evento em si, para a qual a ocorrência contribui. Já os consequentes se referem a um conjunto de fatores desencadeados assim que confirmado o fenômeno em questão^(8)^.

Enfatiza-se que algumas etapas do Modelo de Análise do Conceito são equivalentes e ocorrem de forma simultânea às etapas da revisão integrativa da literatura, como seleção do conceito; determinação dos objetivos da análise conceitual; e identificação dos possíveis usos do conceito^(8)^. Destaca-se que, inicialmente, para realização da análise do conceito, selecionou-se o conceito Juventudes. Nesta etapa, os objetivos foram: realizar a análise de conceito do termo Juventudes para identificar os usos (definições), os atributos críticos, os possíveis antecedentes e os consequentes de Mobilidade. Deste modo, tendo como objetivo principal encontrar uma estrutura inicial para o conceito que permitisse construir uma definição clara e estabelecer os elementos causais (antecedentes) e possíveis expressões e resultados associados à ocorrência daquele conceito. Além disso, o trabalho visa apresentar discussão mais específica dos elementos encontrados, de modo a minimizar a necessidade das outras etapas.

Para realização das etapas propostas, realizou-se revisão integrativa da literatura que seguiu as cinco etapas sugeridas: identificação de tema ou questionamento da revisão integrativa, amostragem ou busca na literatura, avaliação dos estudos incluídos, interpretação dos resultados e síntese do conhecimento evidenciado nos artigos analisados na revisão^(9)^.

Para elaboração da questão de pesquisa, utilizou-se da estratégia PICO (acrônimo para *patient, intervention, comparison, outcomes*)^(10)^. O primeiro elemento da estratégia (P) se refere a adolescentes, jovens e juventudes; o segundo (I) se refere ao período de vida vivenciado; quarto elemento (O) diz respeito aos grupos sociais. Neste estudo, não se utilizou do elemento comparação (C), porque esta pesquisa não visa realizar comparação entre as intervenções. O uso da estratégia PICO se mostra eficiente na recuperação efetiva de evidências, algumas bases possuem interface para inserção direta dos quatro componentes da estratégia PICO, como a MEDLINE/ PubMed. Quais os atributos, antecedentes e consequentes relacionados ao conceito de juventudes identificados na literatura?

Após o levantamento dos conceitos principais na literatura, parte-se para determinação dos atributos definidores, ou seja, a reunião de atributos que são mais frequentemente associados ao conceito. A ideia de atributo compreende aquilo pelo qual uma substância é conhecida ou da teoria dos atributos daquilo que exprime a essência da substância ou conceito. Na etapa de identificação dos antecedentes e consequentes do conceito, ocorre, então, a verificação dos eventos que ocorrem antes do fenômeno de interesse (antecedentes), sendo necessários para a ocorrência destes, assim como os que acontecem posterior ao conceito e resultam da presença do fenômeno (consequentes)^(8)^.

### Período e local do estudo

Desenvolveu-se busca via Biblioteca Virtual em Saúde (BVS), que incluiu as bases Literatura Latino-Americana e do Caribe em Ciências da Saúde (LILACS), Literatura Internacional em Ciências da Saúde (MEDLINE), Base de Dados de Enfermagem (BDENF) e Índice Bibliográfico Espanhol de Ciências da Saúde (IBECS).

Utilizou-se da combinação dos Descritores em Ciências da Saúde (DeCS): juventude; jovem; adolescência. Destaca-se que os descritores foram combinados por meio do operador booleano *AND*, tendo como combinação chave: “juventude” *AND “*juventudes” *AND “jovem”* AND *“adolescência”.* A busca ocorreu de forma pareada pelos pesquisadores, sendo executada em maio e junho de 2023. Os dados foram coletados por meio de instrumento estruturado, contendo a caracterização dos estudos, atributos, antecedentes e consequentes identificados na literatura quanto ao conceito de juventudes.

### Critérios de inclusão e exclusão

Incluíram-se os estudos em português, espanhol ou inglês, sendo selecionados os artigos originais, teóricos, de reflexão e artigos de revisão, disponíveis na íntegra. Como critérios de exclusão, consideraram-se artigos de opinião, cartas ao editor, dissertações e teses, pois a literatura cinzenta é a literatura não controlada por editores científicos ou comerciais. Excluíram-se, também, aqueles que não subsidiaram melhor entendimento da temática em questão. Optou-se pela restrição temporal a partir de 1990, por se tratar da data na qual o termo foi incorporado.

Inicialmente, encontraram-se 106 artigos. Dentre os artigos investigados, excluíram-se 24, por se tratarem de textos indisponíveis ou teses e dissertações. Enquanto 13 foram excluídos por se tratarem de duplicatas, sendo selecionados 69 para a leitura do título e do resumo, após essa etapa, eliminaram-se quatro, por não estarem relacionando jovem/juventudes nos títulos ou resumos. Assim, realizou-se a leitura na íntegra de 65 artigos, sendo excluídos 43 que não apresentavam relação direta com a conceituação de Juventudes. Ao final, selecionaram-se 22 artigos selecionados. Destes, 19 estão indexados na base LILACS, enquanto dois estão na base BDENF e um, na IBECS.

### Análise dos resultados

A coleta de dados foi orientada por formulário desenvolvido pelos pesquisadores com expertise em trabalhos com adolescentes e juventudes, assim como revisões na literatura para extração das seguintes variáveis: base de dados; idioma; ano de publicação; país de desenvolvimento do estudo; objetivo do estudo; desenho metodológico; conceitos centrais sobre a temática. Assim, os dados foram organizados e digitalizados em planilhas eletrônicas, disponíveis no programa *Microsoft Excel*® 2017. Realizou-se a síntese descritiva dos achados, a fim de clarificar os conceitos referentes aos dados encontrados.

## RESULTADOS

A seleção dos artigos foi realizada mediante a busca nos bancos de dados presentes na Biblioteca Virtual da Saúde (BVS), atingindo o total parcial de 22 artigos. O caminho percorrido durante essa busca segue sintetizado na Figura 1.

**Figura 1:** Fluxograma do processo de seleção dos estudos para revisão integrativa, adaptado de PRISMA-ScR, 2024

Dentre a amostragem de estudos, apresentada no Quadro 1, verificou-se que a maioria foi desenvolvida na área de conhecimento da Psicologia (n=9), seguida da Sociologia (n=4), Enfermagem (n=4) e Educação Física (n=2). Outras áreas com menos trabalhos, como o caso da Filosofia (n=1), Terapia Ocupacional (n=1), Serviço Social (n=1). Não houve abordagem expressiva sobre o conceito juventudes na área da Enfermagem, reafirmando a necessidade de defini-lo.

**Quadro 1:** Descrição dos estudos incluídos da revisão integrativa, segundo título, autores/ano de publicação, nível de evidência, delineamento, número de participantes e cenário, intervenções empregadas nos cuidados paliativos e desfecho, 2024

ID	Títulos	Autores/Ano de Publicação	Revista/Base de Dados	Objetivos	Síntese do conceito de juventude/juventudes
A1	Reflexões acerca da prática clínica do enfermeiro junto às juventudes^(6)^	Castro Júnior AR, et al., 2020	Cultura de Los Cuidados/IBECS	Refletir sobre a prática clínica do enfermeiro junto às juventudes.	O entendimento sobre os vários fatores que envolvem a vida do jovem faz o termo juventude exigir a pluralização, utilizando, assim, “juventudes” para determinar diversas realidades que se encontram esses grupos juvenis.
A2	Percursos metodológicos para a apreensão de universos de adolescentes e jovens: um enfoque sobre a questão das drogas^(11)^	Pereira PE, Malfitano APS, 2012	Journal of Human Growth and Development/LI LACS	Discutir o uso de metodologias de pesquisa participativas na apreensão dos universos juvenis.	Existem diferentes maneiras de se vivenciar a juventude, a depender do contexto social, histórico, econômico, político; do gênero, classe social, etnia, local de residência (rural ou urbana, no centro ou na periferia). Logo existem juventudes.
A3	Mapa das juventudes de Santo André, SP: instrumento de leitura das desigualdades sociais^(12)^	Yonekura T, et al., 2010	Revista Saúde Pública/LILACS	Analisar as desigualdades sociais de jovens moradores em área urbana pelo mapeamento de dados sociodemográficos e econômicos.	A perspectiva da saúde coletiva compreende a juventude a partir das categorias classe social e geração, submetendo a leitura da realidade contemporânea dos jovens à condição de classe. Há, portanto, várias juventudes, e embora tenham em comum a condição geracional, acabam por vivenciá-la de diferentes formas, de acordo com a inserção das famílias e da sociedade.
A4	Consulta de enfermagem no cuidado ambulatorial às juventudes^(13)^	Castro Junior AR, et al., 2019	Revista de Enfermagem da Universidade Federal de Pernambuco/BD ENF	Identificar as vidências científicas sobre a consulta de Enfermagem no cuidado ambulatorial às juventudes.	Na área da saúde, uma imprecisão conceitual sobre a adolescência, os jovens e a juventude, partindo, primeiramente, da arbitrariedade na demarcação do limite etário. Verifica-se, ao se considerar os documentos legais sobre adolescência e juventude, que mesmo esses não apresentam definições unificadas sobre a época da vida definida por esses termos.
A5	*Género y juventudes Argentinas: ¿de qué estamos hablando cuando hablamos de “el problema del embarazo adolescente”?* ^(15)^	Duran, ABD, Muro MM, 2020	Sexualidad, Salud y Sociedad Revista Latinoamericana/ LILACS	Examinar a questão da gravidez na adolescência como um problema, por meio de um percurso histórico, pelas fontes jornalísticas e por um corpus jurídico.	A conceituação de “juventude” no plural, para enfatizar que existem várias formas de ser jovem, ou seja, que longe de ser um fenômeno homogêneo, “leva a considerar diferentes adolescentes, pautados por diferentes processos sociais e por variados marcadores sociais de diferença, como classe social, gênero e raça”.
A6	Juventudes e participação: compreensão de política, valores e práticas sociais^(16)^	Mesquita MR, et al., 2016	Psicologia e Sociedade/LILA CS	Compreender as diferentes formas de inserção e atuação de jovens em diferentes grupos e movimentos, considerando, principalmente, as compreensões de política e os valores e concepções que norteiam as práticas.	As juventudes são ao mesmo tempo uma condição social e um tipo de representação que não mais diz respeito apenas a uma determinada fase da vida, relacionada a uma faixa etária. Enfatiza-se que o grupo não pode ser caracterizado como homogêneo pelo fato dos membros possuírem apenas a idade em comum.
A7	*Contextualización teórica al tema de las juventudes: una mirada desde las ciências sociales a la juventud^(17)^*	Alvarado SV, Posada JEM, Gaviria DAM, 2009	Sexualidad, Salud y Sociedad Revista Latinoamericana/ LILACS	Apresentar abordagem às teorias sociológicas contemporâneas, a partir do tema da juventude, com base em alguns conceitos, a partir dos quais as ciências sociais, e especificamente a sociologia, têm compreendido a dinâmica juvenil.	O entendimento de juventudes envolve um diálogo de discursos, e que estes obedecem a condições espaço/tempo específicas, é pertinente propor possível percurso temático que reflita sobre os imaginários simbólicos produzidos e produtores de percepções e autopercepções das juventudes e, portanto, transcendem a mera localização psicoevolutiva, em determinada faixa etária.
A8	A percepção das juventudes a respeito dos seus processos de identidades e suas apropriações para além do jogador Neymar^(18)^	Feltes AF, Sanfelice GR.	Movimento/LIL ACS	Analisar e interpretar a relação entre a construção de identidades e a percepção das juventudes acerca das formas de apropriação referentes à figura do jogador Neymar.	Diferentes grupos sociais utilizam a representação para construir as próprias identidades e as identidades dos outros grupos, mesmo que essa identificação seja temporária. As juventudes constroem os traços identitários, por meio das referências em comunidades e não querem estar alocados a estereótipos.
A9	Juventudes e medida socioeducativa: o olhar dos agentes pedagógicos sobre as práticas de lazer^(20)^	Tretin DFL, et al., 2018	Pensar a Prática/LILACS	Investigar a perspectiva dos agentes pedagógicos sobre as práticas de lazer de adolescentes em cumprimento de medida socioeducativa na cidade de Campinas-SP, Brasil.	Entende-se que a juventude é a fase da vida em que as experiências e situações cotidianas têm grande influência sobre a formação integral do indivíduo. Ademais, o termo “juventudes” caracteriza um estrato social com certas características: “uma realidade palpável que tem sexo, idade, raça, fases, de suas condições materiais e sociais”.
A10	Juventude em Foco: Representações Sociais da Juventude na Folha de S.Paulo^(21)^	Rezende JWR, Espindula DHP, 2020	Psicologia: Ciência e Profissão/LILAC S	Investigar as representações sociais da juventude em matérias do jornal Folha de S. Paulo.	A concepção de Juventudes apresenta a ideia da existência de uma pluralidade de juventudes, ao atentar para o fato de que a mídia se refere a esse período, por meio de um conjunto de aspectos não somente biológicos, como também culturais e sociais, experiências de vida e momentos compartilhados.
A11	Jovens, imagens de si e a cidade: discursos em movimento^(22)^	Levitan D, et al., 2009	Journal of Human Growth and Development/LI LACS	Compreender as relações existentes entre juventude e cidade, bem como os sentidos que permeiam este momento da vida para algumas jovens.	Juventudes, no plural, e não de juventude, no singular, para não esquecer as diferenças e desigualdades que atravessam esta condição. O uso do plural indica o reconhecimento de que a juventude é uma condição existente em variados grupos sociais, mas que pode ser significada distintamente por cada um deles, enfatizando os diferentes modos de vivência de tal momento.
A12	*Hacia un entendimiento del aprendizaje en entornos digitales-Implicaciones para la educación^(23)^*	Morales-Barreira MCM, 2018	Sexualidad, Salud y Sociedad Revista Latinoamericana/ LILACS	Analisar as características da aprendizagem, a partir das competências digitais que os jovens do ensino superior desenvolvem em ambientes socioculturais digitais não escolares.	A pluralização do termo implica perspectiva epistemológica da juventude, baseada no reconhecimento da heterogeneidade como eixo de múltiplas construções subjetivas que não podem ser subsumidas em uma identidade fechada. Desse ponto de vista, a categoria juventude tem duas implicações importantes: a erosão da suposta identidade da juventude e a necessidade de historicizar a produção de subjetividades juvenis.
A13	Encontros com a diferença na formação de profissionais de saúde: juventudes, sexualidades e gêneros na escola^(24)^	Silva CG, Borba PLO, 2018	Saúde e Sociedade/LILA CS	Discutir a forma como gênero e sexualidades são significados por jovens secundaristas, com base nas reflexões advindas do projeto de extensão universitária “Juventudes e funk na Baixada Santista*”*.	Nas discussões realizadas, valorizam-se as possibilidades de identificar e compreender intersecções entre os marcadores sociais das diferenças que constituem jovens. Como ferramenta metodológica e analítica, a interseccionalidade permite decodificar irregularidades e regularidades que se inscrevem nos sujeitos das juventudes nos territórios que os constituem.
A14	Os projetos de vida dos jovens da maior favela carioca, a Maré^(26)^	Santos SRR, 2018	Revista de Psicologia/LILA CS	Apresentar os projetos de vida educacional de 89 jovens moradores da favela da Maré, no Rio de Janeiro, Brasil, que se encontravam na última série do Ensino Médio.	“Jovem” e “juventude”, apesar de serem tratadas como sinônimos, “jovem” se refere sempre ao sujeito e “juventude” ao tempo do sujeito. A juventude não é apenas um período de alterações; ela se constitui, também, como fase de planejamento, de construção do futuro.
A15	O lugar das juventudes nas produções científicas da Estratégia Saúde da Família: uma revisão sistemática de literatura^(28)^	Benício LFS, et al., 2019	Textos e Contextos (Porto Alegre)/LILACS	Desenhar um diagrama das tramas de saber-poder em torno das juventudes no campo da saúde.	Na sociedade capitalística, o jovem é construído a partir de uma perspectiva desenvolvimentista que o coloca como um ser em construção, cujos comportamentos são produtos de mudanças físicas e psíquicas. Dentro das várias juventudes, são adicionadas a violência e a criminalidade, o que produz a noção de “juventude perigosa”.
A16	*Miradas docentes sobre los jóvenes y las jóvenes como «otros indeseables» en secundarias universitarias en La Plata* ^(30)^	Piero ED, 2019	Revista Latinoamericana de Ciencias Sociales, Niñez y Juventud/LILAC S	Apresentar a visão dos professores sobre os jovens como “outros indesejáveis” nas escolas secundárias universitárias de La Plata.	Nas proposições mais biológicas ou demográficas, homens e mulheres jovens são definidos com base na idade biológica. Pelo contrário, pesquisas recentes têm destacado o surgimento de uma nova “condição juvenil”, em um contexto de profundas mudanças sociais, econômicas e culturais.
A17	Primaveras e jovens tardes: a memória saudosa da mocidade/juventude na música popular brasileira^(31)^	Nascimento ARA, et al., 2010	Memorandum/LI LACS	Identificar, em correlação, os elementos saudosamente recordados relativos ao período da juventude/mocidade presentes em letras de canções brasileiras compostas e/ou gravadas a partir de 1927.	Admite-se a existência de diversas representações de juventude, coexistindo em uma mesma sociedade. Aqui, deve-se considerar novamente que o compartilhamento de determinado significado não necessariamente pressupõe a percepção da “juventude” como uma categoria unívoca.
A18	Produção social, histórica e cultural do conceito de juventudes heterogêneas potencializa ações políticas^(32)^	Trancoso AER, Oliveira AAS, 2014	Psicologia e Sociedade/LILA CS	Produção social, histórica e cultural do conceito de juventudes heterogêneas potencializa ações políticas.	As juventudes “são construções sociais, são ‘classes de idade”’ que “apesar de possuírem uma base material biológica, têm diversas representações históricas”. O termo juventudes não compreende apenas as mudanças corporais e hormônios tidas pela idade, mas demarcam raízes epistemológicas da cultura.
A19	Programa Nacional de Inclusão de Jovens: possibilidades e Contribuições na Perspectiva dos adolescentes participantes^(33)^	Campos CCA, Paiva IL, 2018	Fracal/Revista de Psicologia/LILA CS	Discutir o processo socioeducativo do Programa Nacional de Inclusão de Jovens, na modalidade Projovem Adolescente (PJA), utilizando como recorte a perspectiva dos adolescentes participantes da cidade de Natal/RN.	Existe a dicotomia, no que se refere à concepção de juventude. Em virtude da diversidade de situações existenciais que afetam os indivíduos nessa etapa do ciclo de vida, tem sido recorrente a importância de se tomar a ideia de juventude e adolescência em sentido plural - juventudes e adolescências.
A20	Uma caderneta que produz ‘saúde´ e ‘adolescentes´^(34)^	Hüning SM, Luz Valeska CM, 2011	Arquivos Brasileiros de Psicologia/LILA CS	Problematizar a Caderneta de Saúde do Adolescente, produzida pelo Ministério da Saúde.	Ao estudar a vida do adolescente, podem ser percebidas e estudadas como um conjunto social diversificado, identificado por diferentes culturas juvenis. Ao utilizar-se o termo juventudes, no plural, busca-se compreender a complexidade e heterogeneidade.
A21	Uma Promessa Civilizatória Perversa: as políticas públicas e juventudes na era neodesenvolvimentista^(35)^	Scherer GA, Gershenson B, 2016	Textos e Contextos (Porto Alegre) LILACS	Apresentar o contexto de (des)proteção social vivenciado pelas juventudes brasileiras, revelando as principais expressões da questão social que impactam nesse segmento social.	Considera-se o termo “juventudes” no plural como forma de indicar sua multiplicidade, partindo do pressuposto de que é necessário ter em conta uma série de particularidades que compõem esse segmento como raça, classe social, território, entre outros elementos.
A22	Jovens em webrádio: representações sociais sobre papilomavírus humano^(36)^	Araújo AF, et al., 2019	Revista de Enfermagem da Universidade Federal de Pernambuco/BD ENF	Aprender as representações sociais de jovens escolares acerca do papiloma vírus humano, por meio de uma webrádio.	Desenvolvem-se as juventudes nas perspectivas pessoal e social, como sujeitos de direitos e como atores com papel especial de transformação da realidade, marcadas centralmente por processos de desenvolvimento, inserção social e definição de identidades diversas.

Quanto ao idioma, houve predominância de pesquisas publicadas na língua portuguesa (n=18), tendo em vista a maior parte da amostra ter sido produzida no Brasil (n=18). Houve ainda artigos no idioma espanhol (n=4), sendo distribuídos entre os países: Colômbia (n=2) e Argentina (n=2), demonstrando a utilização do termo internacionalmente.

Ao considerar os aspectos metodológicos, prevaleceu abordagem qualitativa, em especial o tipo de estudo exploratório (n = 10), seguido de reflexivos (n=6), revisão bibliográfica (n=3) e documental (n=1). Houve pequena expressão de artigos quantitativos (n=1), correspondendo a um estudo ecológico (n=1). No que se refere aos elementos abordados conforme fenômeno, antecedentes, atributos e consequentes, estes estão apresentados em categorias e subcategorias de análise, conforme Figura 2.

**Figura 2:** Antecedentes, atributos e consequentes do conceito de juventudes, 2024

## DISCUSSÃO

Identificaram-se, nesta análise, diversos componentes que definem Juventudes, dando destaques a elementos que compõem os antecedentes, atributos e consequentes, mediante a análise. Acredita-se que esse fato esteja vinculado à grande amplitude do entendimento do que seja as juventudes, o qual vem se modificando ao longo do tempo, sobretudo na compreensão de cada área do saber. Conforme exposto na síntese, a Enfermagem ainda discute pouco sobre o assunto, muitas vezes, citando fontes das ciências sociais. Dentre os estudos analisados nesta pesquisa, a maioria associou o jovem à concepção etária, dentre outros elementos mais físicos, enquanto as juventudes configuram as relações que esses jovens têm com os diversos componentes da vida. Logo, há a necessidade da implementação de conceitos mais elaborados sobre as juventudes, corroborando manuscritos que discorrem as ações de enfermeiros na prática clínica.

Os estudos levantados destacam que o fenômeno das juventudes não pode seguir relacionado, hegemonicamente, ao modelo etário de divisão. Neste sentido, a discussão dos achados aborda de forma sucinta os elementos que compõem os antecedentes, atributos e consequentes do termo juventudes, relacionando, assim, esses componentes com a compreensão dos autores sobre definições e abordagens na vida das juventudes.

### Antecedentes do conceito “juventudes”

Os antecedentes compreendem os determinantes do conceito, aquilo que antecede o evento e o surgimento do desfecho. Assim, os resultados buscam apresentar que o conceito de juventudes é resultante de situações relacionadas às vivências dos grupos juvenis, desencadeando nesses grupos o plural da compreensão da juventude.

A construção do conceito de juventudes perpassa pela compreensão social que envolve diversos fatores. Compreende a definição de jovem como um indivíduo com idade entre 15 e 29 anos, entendendo que essa conceituação engloba as modificações no campo biológico com a maturação das características físicas, expressão da sexualidade e vivências sexuais quanto à orientação sexual e identidade de gênero e expressões da sexualidade, que não se restringem ao ato sexual, mas abarcam ainda expressões e práticas das juventudes em se relacionar. Cabem ainda nesse período, as intensas modificações no campo psicológico, a compreensão enquanto sujeito, conflitos pessoais causados pelas modificações e pela maturação^(11)^.

Esse momento de modificações do jovem impulsiona o surgimento de dúvidas sobre a transição do corpo, assim como nas relações. Junto a essas modificações, surge a busca pela própria identidade, tendo como base as vivências familiares, como também a relação com amigos (pares). Essa relação influencia a construção de laços e a busca por aceitação em grupos sociais em que se sinta pertencente^(12-13)^.

A compreensão sobre o conceito juventudes compreende as múltiplas referências que incidem na vivência do jovem, questões de gênero, classe social e etnia são discutidas com frequência, ao se tratar dessa definição. Jovem, juventude e a pluralização destas podem ainda estar enraizadas em diversos estereótipos que tendem a definir de forma incorreta esse grupo, muitas vezes, colocando como “figura problema”^(14)^.

Essa multiplicidade de fatores e vivências sobre um mesmo recorte populacional reforça a dinâmica sobre a permanente evolução no conceito de juventude, dando sentido à pluralização do termo, tendo assim “juventudes”, pois não é possível definir essa palavra sem considerar toda construção social, política e econômica que incide sobre esses sujeitos^(11)^.

Na definição de juventudes, há espaço para compreensão das desigualdades sociais e como esse fator impacta na população, sobretudo, na condição de acesso aos bens e serviços, a exemplo da saúde, educação e segurança. Essa dinâmica facilita o reconhecimento de uma heterogeneidade no grupo, uma vez que essa dinâmica social determina onde e como vivem as pessoas, assim como influenciam as relações e as influências em que se tem acesso^(15)^.

A compreensão sobre a dinâmica social é importante para a definição do conceito de juventudes, assim como o entendimento sobre o tempo histórico que se considera essa definição. A figura da juventude tem ampla discussão na história, mas sempre distanciando o conceito de qualquer pretensão homogeneizadora sobre a população^(12,15)^.

As distintas formas de contato e participação política podem determinar no território (país, estado), a forma de como é vista essa população, assim como as ações direcionadas a esses jovens, no concernente à saúde, à segurança, ao esporte, ao trabalho, dentre outros aspectos que influenciam as oportunidades de crescimento dos sujeitos. Torna-se importante reconhecer a existência de múltiplas formas de organização das juventudes e a inserção social destas em grupos de constantes transformações, para assim definir o que são juventudes e o alcance destas enquanto conceito^(16)^.

### Atributos do conceito “juventudes”

Os atributos compreendem as propriedades do conceito, sendo elementos que expressam as características principais do constructo. Esses atributos podem se desvelar como relações sociais, influências do meio, dentre outras características, a depender do conceito e do objeto central. Assim, apresentam-se aqui os sentidos da pluralização do termo e a respectiva repercussão coletiva. O processo de identidade é identificado como a expressão daquilo que une os indivíduos conforme as escolhas individuais e coletivas e dão sentido àquele coletivo. Assim, a compreensão sobre o conceito de juventudes está cada vez mais atrelado ao compartilhamento de vivências e experiências que colocam o sujeito dentro de um território de experimentações que o confere a noção de fazer parte daquele lugar, daquele grupo^(11)^.

Compreende-se, por meio dos achados, que as juventudes se forjam a partir das colocações sociais e geracionais (tempo histórico em que se insere), porém, as discussões são bem fortes sobre o compartilhamento de vivências. As várias juventudes, mesmo diante do mesmo tempo histórico, têm as próprias vivências, e se organizam em diferentes culturas juvenis, identificando-se em grupos diferentes^(12,17)^.

As identidades das juventudes são formuladas por meio de vivências, assim como práticas coletivas de interesses em comum, que culminam com a ideia de “estar junto” e ao “ser parte”^(18)^. Com a formulação coletiva dos sentidos que unem os grupos sociais e que tem essa ideia de identidade de grupo, é comum ter afinidade pela união de expressões culturais e raciais comuns. Estar próximo de alguém que se identifica, gera o acolhimento para o sujeito. Embora haja delimitações para a condição juvenil, a vivência de ser jovem requer a pluralização^(19-20)^.

Conforme a ideia de construção coletiva e de todas as expressões culturais que envolvem aquele jovem, evidenciadas nos textos, o conceito juventudes deixa de ser limitado a uma fase do desenvolvimento humano e passa a abranger um modo de vida, de identificação dentro de um grupo que se conecta por uma interpretação de mundo semelhante^(21)^.

A partir da compreensão dos diferentes modos de se relacionar no decorrer da vida, conforme os contextos sócio-históricos particulares, a concepção plural de juventude rompe a lógica universal e rígida, que não contempla todas as vivências desse grupo. O plural serve, então, para lembrar as diferenças e desigualdades que atravessam esta condição. O modo de compreender o mundo dentro dos diferentes grupos sociais contém sentidos singulares, enfatizando os diferentes modos de vivência ou como essas vivências incidem sobre os sentimentos compartilhados pelo grupo^(22)^.

É possível identificar, mediante os achados da literatura, a pluralização das juventudes sob uma perspectiva epistemológica, tendo como eixo as construções subjetivas que não podem ser reduzidas a uma determinação etária. Invisibilizar as diferentes condições e os modos de ser jovem, compreende a exclusão de tudo o que não se enquadra no identitário da juventude, desconsiderando sentidos e sentimentos^(23)^.

Essa conjuntura de grupo é a construção de uma rede social produtora de subjetividades que conectam sujeitos e permitem identificar sentimentos, como a alegria de ser jovem, a rebeldia com as transformações e a impulsividade da idade. Assim, compreende uma dinâmica relacional dos sujeitos que produzem conexões e apoio no contexto de vivências^(24)^.

### Consequentes do conceito “Juventudes”

Os consequentes evidenciam o desfecho do conceito analisado, neste sentido, revelam como o conceito se expressa e o que esse fenômeno ocasiona para a população, no caso, as juventudes. Evidenciaram-se como principais consequências: a autoimagem, o desenvolvimento intelectual, os comportamentos para a saúde, as diferenças nas oportunidades de crescimento individual, as dinâmicas na busca por serviços de saúde, o planejamento e a construção de futuro.

A autoimagem discutida nos achados revela o modo de ser e viver, os valores vigentes e as relações que se estabelecem na sociedade. É formada por meio das vivências cotidianas dos jovens, condição que parte do arranjo individual e compõe uma compreensão sobre si, a partir da fusão dos padrões sociais e das relações construídas. Destaca-se a relevância crucial da autoimagem na construção da identidade^(25)^.

A construção da identidade juvenil é destacada na literatura como intrínseca ao desenvolvimento intelectual. Este ocorre a partir das vivências pessoais e sociais, sendo determinadas por habilidades desenvolvidas desde a infância até a vida adulta. Neste sentido, a família, a escola e o contexto social em que os jovens estão inseridos têm papéis fundamentais^(26)^.

Conforme o contexto social de inserção dos jovens, evidenciadas nas realidades trazidas nos artigos, pode-se inserir diferentes oportunidades diante dos núcleos socioeconômicos. A convivência com o meio e as influências destes podem direcionar práticas e escolhas que interligam o desenvolvimento da vida e das oportunidades desses jovens, quanto ao trabalho, à educação e às condições sociais. Nem tudo é determinado pela interação social, mas esta liga o modo de produzir vivências e enfrentar as oportunidades e adversidades. Existem estigmas relacionados aos jovens de camadas populares, em maioria, negros e pobres, moradores de periferias dos centros urbanos, taxados de violentos e delinquentes, cujas situações forjam iniquidades que deixam esse grupo juvenil em diferentes condições e menores oportunizações de melhoria de vida^(27)^.

Os artigos identificam que os modos de viver, a ideia do jovem “invunerável”, a formação das influências são importantes fatores a se considerar quando se observa a busca por serviços de saúde. A formação do jovem influencia em pouca procura pelos serviços, quando procurados, apresentam resistência e dificuldade para acolher as demandas juvenis, considerando os comportamentos de saúde desse público, que podem ser seguros ou de risco. Questões como mudança corporal, desenvolvimento intelectual, formação de identidade pessoal e convivência com pares são fatores que influenciam a primeira experiência sexual, o uso de álcool e outras drogas, sendo o álcool considerado fator de risco para as práticas sexuais inseguras e a aquisição de infecções sexualmente transmissíveis^(28)^.

Nos diversos contextos sociais de inserção do jovem, a juventude é destacada nos conceitos apreendidos na literatura como uma fase de planejamento, de construção de mundo, sendo considerada etapa de transição para a vida adulta. Na Idade Medieval, essa fase era capitaneada por rituais. Nos dias atuais, esse processo ocorre por meio dos grupos sociais aos quais os jovens estão inseridos, como a família, a escola, a igreja, o círculo de amizades^(24,28-29)^.

A literatura destaca ainda que a amizade, a possibilidade do encontro e a partilha dos afetos produzem e realçam a ideia de pertencimento ao grupo, reforçam os ideais comunitários, culturais e políticos, bem como possibilitam a construção de projetos comuns frente aos inúmeros desafios vividos. Estes valores impulsionam as práticas e os fazem resistir diante das dificuldades enfrentadas, em um sentimento de superação, renovação e aprendizado^(16,30-32)^.

Salienta-se que os valores compartilhados pelos jovens são de diferentes ordens. No entanto, tendo o desejo pela transformação como valor maior, eles constroem noções de coletividade e comunidade que os ajudam a se reconhecer como agentes importantes de mudanças, tão necessárias para materializar os anseios de uma sociedade melhor. Além disso, eles compartilham valores de ordem mais subjetiva, como a amizade e o prazer, por exemplo, que podem se firmar como dispositivos importantes para a organização dos grupos e o processo de subjetivação política^(16,33-35)^.

Como consumidores de bens e serviços, no quesito saúde, possuem dificuldade de acesso, sendo as necessidades reduzidas ou desconsideradas no âmbito das singularidades. No campo da saúde, ainda há lacunas de estratégias que permeiam um cuidado que compreenda as representações das juventudes e das respectivas particularidades, representando desafio aos serviços e profissionais^(6,29)^. O preenchimento dessas lacunas pode beneficiar a compreensão sobre a prática do cuidado clínico a essa população, compreendendo as necessidades desta e atuando de modo a considerar as limitações e possibilidades para com esse público^(20,29)^.

Frente ao exposto, o conceito de juventudes compreende a expansão do conceito de jovem, considerando as mudanças corporais e psicológicas que acontecem no período de 15 a 29 anos, mas, sobretudo, os fenômenos responsáveis pela construção da personalidade, como sexualidade, identidade de gênero e as relações sociais.

A construção das juventudes envolve a nuance das relações sociais, seja com familiares ou os pares, que influenciam como o sujeito individualmente constrói a ideia de ser social e suas relações, a aproximação com esses grupos trazem, muitas vezes, estereótipos que cristalizam a imagem do jovem. Contudo, a compreensão sobre o conceito ainda está ligada à construção social, política, econômica e cultural em que esse jovem tem vivência e que influenciam diretamente em acesso aos bens e serviços (saúde, segurança, educação).

As juventudes se organizam em grupos juvenis com características comuns ao grupo muitas vezes unido pela partilha de vivências que tem a ver com o momento histórico e como este influencia na vida do jovem, desde a implementação de políticas públicas até a compreensão sobre o que é ser juventudes plurais.

Por fim, conceituam-se juventudes como grupo social com identidade grupal própria, no qual as questões sociais, culturais e raciais influenciam a interpretação de mundo, o compartilhamento de vivências e pertencimento ao território, sendo caracterizado por sentimentos de alegria, rebeldia, impulsividade e agressividade.

### Limitações do Estudo

Essa pesquisa possui limitações que devem ser consideradas. Inicialmente, compuseram esta análise conceitual apenas estudos disponíveis eletronicamente, em determinadas bases de dados, o que pode ter ocasionado a exclusão de pesquisas que contemplam outras perspectivas sobre as juventudes. Destaca-se também como limitação do estudo o uso exclusivo de um único portal para realizar a busca de dados, o que pode restringir a abrangência da pesquisa e limitar a variedade de informações disponíveis.

### Contribuições para a Área

Os achados promovem o entendimento da amplitude da compreensão sobre os grupos das juventudes e a magnitude das possibilidades de adoção de estratégias do enfermeiro. A análise de conceito possibilita incluir o fenômeno em programas de formação da equipe de enfermagem. A ampliação da compreensão sobre esse grupo populacional facilita a compreensão de enfermeiros em diferentes realidades, possibilitando que a equipe multidisciplinar trabalhe para atuação desde o campo da promoção da saúde, redução de danos e minimização de agravos, entendendo que são necessárias diversas estratégias para alcance desse público. O esforço em analisar o conceito consiste o primeiro passo para compreender os sujeitos e os fenômenos sociais que envolvem as vivências deles, de modo a ampliar o campo de atuação do enfermeiro.

## CONSIDERAÇÕES FINAIS

O conhecimento dos antecedentes, atributos e consequentes auxilia na compreensão do conceito de juventudes na área da saúde, especialmente para a Enfermagem. Essa compreensão delimita as informações importantes sobre a formação dessas juventudes e os grupos juvenis, expandindo a compreensão para além da faixa etária e compreendendo os processos que interferem na formação dessas culturas juvenis. Haja vista as ponderações ora expostas, os artigos analisados desvelam o panorama a respeito do conceito “juventudes”, por meio do qual se confirmou a complexidade proveniente da origem multifatorial do fenômeno em estudo. Destaca-se ainda que, mesmo diante do vasto uso do termo juventudes, não se observa consenso na literatura sobre ele, evidenciando, assim, divergentes definições que não subsidiam adequadamente o cuidado de enfermagem às juventudes.

A análise do conceito evidenciada por este estudo pode servir de suporte para uma assistência de enfermagem às juventudes, considerando a pluralidade e heterogeneidade desse público, visando ações direcionadas às reais necessidades dos jovens, tornando o cuidado qualificado e eficaz. Compreender o conceito, oportuniza o agir com essa população jovem, em diferentes contextos e realidades, proporcionando suporte teórico aos enfermeiros nessa atuação.
